# Pain Catastrophizing and Its Relationship with Health Outcomes: Does Pain Intensity Matter?

**DOI:** 10.1155/2017/9762864

**Published:** 2017-02-28

**Authors:** Carlos Suso-Ribera, Azucena García-Palacios, Cristina Botella, Maria Victoria Ribera-Canudas

**Affiliations:** ^1^Department of Basic and Clinical Psychology and Psychobiology, Universitat Jaume I, Castelló de la Plana, Spain; ^2^Ciber Fisiopatología de la Obesidad y la Nutrición (CB06/03), Instituto de Salud Carlos III, Madrid, Spain; ^3^Pain Clinic, Vall d'Hebron Hospital, Barcelona, Spain

## Abstract

Pain catastrophizing is known to contribute to physical and mental functioning, even when controlling for the effect of pain intensity. However, research has yet to explore whether the strength of the relationship between pain catastrophizing and pain-related outcomes varies across pain intensity levels (i.e., moderation). If this was the case, it would have important implications for existing models of pain and current interventions. The present investigation explored whether pain intensity moderates the relationship between pain catastrophizing and pain-related outcomes. Participants were 254 patients (62% women) with heterogeneous chronic pain. Patients completed a measure of pain intensity, pain interference, pain catastrophizing, and physical and mental health. Pain intensity moderated the relationship between pain catastrophizing and pain interference and between pain catastrophizing and physical health status. Specifically, the strength of the correlation between pain catastrophizing and these outcomes decreased considerably as pain intensity increased. In contrast, pain intensity did not moderate the relationship between pain catastrophizing and mental health. Study findings provide a new insight into the role of pain intensity (i.e., moderator) in the relationship between pain catastrophizing and various pain-related outcomes, which might help develop existent models of pain. Clinical implications are discussed in the context of personalized therapy.

## 1. Introduction

The onset and chronification of pain in previously healthy people are known to impact negatively their physical and mental health status [1, 2]. In addition to the effect of pain intensity, extensive research has pointed to the important role of psychological factors when explaining physical disability and mental well-being [3]. For example, pain catastrophizing, which is broadly defined as a tendency to focus excessively on pain and exaggerate its threat value [4, 5], is now considered a key intervention target in psychological therapies into chronic pain together with pain intensity, physical disability, and mood [6].

Pain catastrophizing has been consistently associated with a wide range of health-related outcomes, including pain intensity, interference of pain with patients' life, physical disability, and mental well-being [7–9]. Most importantly, despite its correlation with pain intensity, pain catastrophizing has shown to contribute unique variance to the prediction of health outcomes even when controlling for the effect of pain intensity [10, 11].

Contrary to the previous findings, some authors have argued that the influence of pain intensity on the relationship between psychological factors, such as pain catastrophizing, and health status, has been underestimated for years, especially in relation to Physical Functioning [12]. In line with this idea, a study with healthy individuals revealed that the number of brain areas associated with pain catastrophizing varied as a function of the intensity of induced pain [13], suggesting that pain intensity may act as a moderator of the relationship between pain catastrophizing and brain activity. Specifically, this study showed that, when induced pain was only mild, pain catastrophizing was associated with activation of a large number of brain regions involved in emotional and motor response to pain, pain vigilance, and top-down inhibitory control. By contrast, pain catastrophizing only correlated with a few regions during moderate pain induction. The authors suggested that different levels of pain intensity may compete with pain catastrophizing for attentional resources, which is consistent with previous investigations showing that pain intensity has an intrinsic interruptive nature which is inescapable and attention-demanding [14].

The goal of the present investigation was to explore whether pain intensity is, indeed, an inescapable experience that competes with pain catastrophizing in the prediction of physical and mental health status. If this was the case, the relationship between pain catastrophizing and health outcomes would be reduced when patients experience severe levels of pain intensity. By contrast, the strength of the association would be larger for patients with mild levels of pain. The identification of pain intensity as a moderator in the relationship between pain catastrophizing and health status might have important theoretical and clinical implications.

## 2. Methods

### 2.1. Participants

Participants were recruited at two hospitals. One hundred sixty-four patients were consecutive chronic pain patients attending a Pain Clinic (Vall d'Hebron Hospital). Patients attending this Pain Clinic tend to be characterized by experiencing moderate-to-severe pain [15]. Therefore, to explore our hypothesis at mild levels of pain, we recruited ninety consecutive chronic pain patients attending a Primary Care Center (Clinic Hospital), where pain levels were expected to be lower. In total, 254 pain patients participated in this study. Eligibility criteria included (1) attending the Pain Clinic of the Vall d'Hebron Hospital or the Primary Care Center of the Clinical Hospital; (2) being 18 years of age or older; (3) experiencing pain for at least 6 months; and (4) not having a cognitive or physical disability that would prevent participation. Recruitment started in March 2015 and ended in November 2015.

### 2.2. Procedure

Study design was cross-sectional. Participants were approached by their physician the day of their appointment. If they met the eligibility criteria, participants were asked to give informed consent before completing the measures. The questionnaires were either administered in site at a waiting room or completed at home and returned later, depending on the availability of participants. The same study protocol and procedures were used in both centers. The research was approved by the Ethics Committee of both hospitals.

### 2.3. Measures

#### 2.3.1. Pain Severity and Pain Interference

The Brief Pain Inventory [16] was used to assess average pain intensity and interference of pain with patient's life, as is recommended in clinical guidelines into chronic pain [17]. Item labels range from 0 =* no pain* to 10 =* worst possible pain* for pain intensity and from 0 =* no pain* to 10 =* pain completely interferes* for pain interference.

#### 2.3.2. Pain Catastrophizing

The pain catastrophizing scale of the Coping Strategies Questionnaire (CSQ-C) is composed of 6 items, each with a 0–6 range [18]. Item labels correspond to 0 =* never do that when in pain* and 6 =* always do that when in pain*. The internal consistency of the CSQ-C in the present study was good (*α* = .86), consistent with previous research [19, 20].

#### 2.3.3. Physical and Mental Health

The Short Form-36 Health Survey was used to assess physical and mental health status. The questionnaire assesses eight components of an individual's health, which can be combined into two composite scores of physical and mental health [21]. Physical Functioning (i.e., performance at daily activities), Role Physical (i.e., performance at work), Bodily Pain (i.e., average pain intensity), and General Health (i.e., subjective health experience) have high loadings on the Physical Composite Score. Vitality (i.e., energy as opposed to tiredness), Social Functioning (i.e., interpersonal performance), Role Emotional (i.e., influence of emotions on functioning), and mental health (i.e., psychological well-being) have high loadings on the Mental Composite Score. The use of these two composite scores is recommended because it reduces the number of statistical comparisons [22]. However, the use of the Physical Composite Score in the present study was problematic because it contains a pain subscale, which would contaminate the relationship between the dependent variable (i.e., health outcome) and the moderator (i.e., pain intensity). Therefore, physical health was assessed by means of the subscales (i.e., Physical Functioning, Role Physical, and General Health), excluding Bodily Pain. The use of the physical health subscales as opposed to the composite score is a frequent practice and is not problematic although it increases the number of statistical comparisons. As opposed to the Physical Composite Score, the Mental Composite Score is not contaminated by the presence of pain intensity ratings, so it was used as a measure of overall mental health status to reduce the number of statistical tests. Scales and composite scores in the Short Form-36 have a 0–100 range. High scores reflect better health. The internal consistency of the eight scales in our sample was good (*α* ≥ .82 for all scales), in line with previous research [23].

### 2.4. Data Analysis

First, chronic pain patients recruited at the two assessment sites were compared. A* t*-test for independent samples was used when comparing continuous variables, while a chi-square test was performed for categorical variables.

A series of hierarchical regressions were conducted to explore the moderating effect of pain intensity in the relationship between pain catastrophizing and all study outcomes. Following the recommendations by Baron & Kenny [24], we included the simple effects of pain intensity in the first block. We added pain catastrophizing in the second block. Finally, the interaction term was included in the third block. Average pain intensity and pain catastrophizing were centered by subtracting the mean to avoid multicollinearity problems. Moderation occurs when the interaction term in the third block significantly predicts the dependent variable.

The main goal of the present study was to explore whether the relationship between pain catastrophizing and pain-related outcomes was moderated by pain intensity. However, to control for potential confounders (i.e., two recruitment sites) and important covariates of health, we explored whether moderation also occurred when controlling for recruitment site, age, sex, job status, marital status, educational level, psychopathology, and duration of pain. Covariates were entered in the last block to explore whether the interaction term in block 3 remained significant when controlling for the aforementioned covariates of health status.

Finally, we graphically displayed the relationship between pain catastrophizing and each study outcome across pain groups. As recommended in previous research [25, 26], the following categories were used for pain intensity: mild (1–4), moderate (5-6), and severe (≥7) pain intensity. A regression line was included in each scatterplot to represent the line of best data fit. For each regression, slope, intercept, and explained variance were calculated. Additionally, Pearson correlations were conducted to explore whether the association between pain catastrophizing and the study outcomes varied across each pain intensity group. Bivariate associations and slopes would coincide if study variables had been standardized. However, this was not the case because the use of unstandardized values in the regression provides relevant information on the relationship between predictors and outcomes which cannot be deducted from correlation analyses. Specifically, it informs on the estimated change in the expected value of the outcome for a 1-unit increase in the predictor.

## 3. Results

### 3.1. Sample Description

Participants in the present study were 254 patients (62% women) with heterogeneous chronic pain. Most frequently reported sites of pain were low back (67.5% of participants) and neck (39.3%). Duration of pain ranged from 6 months to 52 years, with a mean of 9.26 years (SD = 9.02) and a median of 7 years. Participants' age ranged from 22 to 90 years old, with a mean of 54.49 years (SD = 12.88). Almost half of participants (49.6%) were married. The majority of the sample (73.6%) had completed more than 12 years of education. A large percentage of patients (61.0%) were not working at the time of assessment. Almost all participants (90.4%) were born in Spain, while the remaining countries of origin occurred at a very low frequency. Approximately 25% of participants reported having a current diagnosis of depression or anxiety.

### 3.2. Comparing Pain Patients from Two Hospitals

A comparison between patients recruited at the Pain Unit and Primary Care patients is shown in supplementary materials (see Tables S1 and S2 in Supplementary Material available online at https://doi.org/10.1155/2017/9762864). Chronic pain patients attending the Primary Care Center were slightly younger, presented a somewhat higher proportion of males, were more likely to be working and to have high educational levels (>12 years of education), and presented lower psychopathology rates. Patients recruited at the Primary Care Center also had lower levels of pain intensity, pain interference, and pain catastrophizing and reported better physical and mental health status.

Of the total sample, 80 patients (31.5%) reported mild levels of average pain intensity, 103 of them presented moderate levels of pain (40.5%), and 71 patients had severe pain (28.0%). The distribution of patients across pain categories was different in both hospitals (*χ*^2^ = 43.71,* p* < .001). Specifically, the proportion of patients presenting severe pain was significantly larger at the Pain Unit (39.6%) when compared to the Primary Care Center (6.7%), whereas the number of patients experiencing mild pain was higher at the Primary Care Center (53.3%, as opposed to 19.5% at the Pain Unit).

### 3.3. Predicting Pain Interference, Physical Health, and Mental Health

Results from the regression analyses predicting pain interference, Physical Functioning, Role Physical, General Health, and the Mental Composite Score are shown in Table 1. In the first block, pain intensity significantly predicted all study outcomes. Similarly, the inclusion of pain catastrophizing in block 2 also contributed significantly to the prediction of all outcomes. Of interest to the present study, the third block revealed a significant moderation effect in the prediction of pain interference (*R*^2^ = 2.1%,* p* < .001), Physical Functioning (*R*^2^ = 1.4%,* p* < .01), Role Physical (*R*^2^ = 1.9%,* p* < .01), and General Health (*R*^2^ = 2.2%,* p* < .01). The moderation was not significant when the Mental Composite Score was the dependent variable.

As a group, the covariates (block 4) added significant variance to the prediction of Physical Functioning, Role Physical, and the Mental Composite Score. However, the inclusion of the covariates only affected the moderation (block 3) when Physical Functioning was the outcome. Specifically, while the regression coefficient of the interaction term was significant in the third block (*β* before including the covariates = .13,* p* = .016), it became nonsignificant in block 4 (*β* after including the covariates = .06,* p* = .258). Further analyses revealed that the inclusion of the recruitment site was responsible for this change. The remaining moderation effects remained significant even when controlling for the covariates.

**Figure 1:**
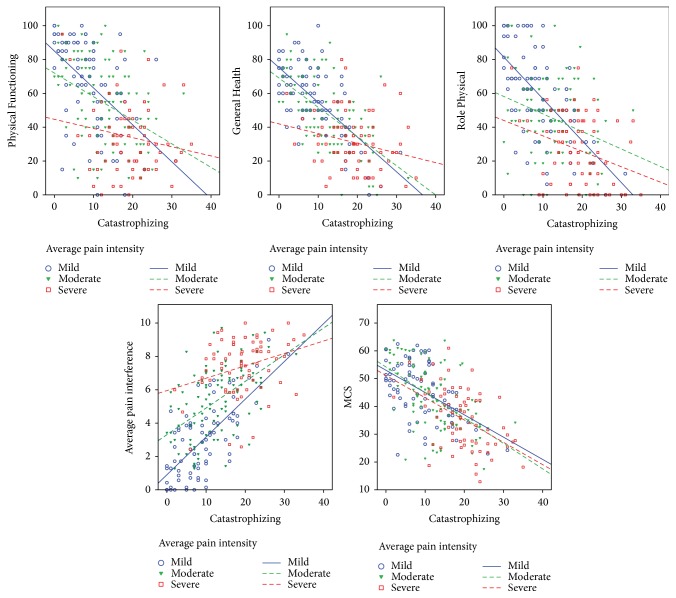
Graphical display of the relationship between pain catastrophizing and study outcomes for mild, moderate, and severe pain intensity.

*Graphical Representation of the Relationship between Pain Catastrophizing and Outcomes across Pain Groups and Exploration of Intercepts, Slopes, Explained Variance, and Bivariate Associations*.  Figure 1 shows a graphical display of the relationship between pain catastrophizing and study outcomes for mild, moderate, and severe pain intensity. A regression line for each pain group was computed. For each pain group, the regression equation was

(1) $$\begin{array}{c} Y=interctep+slope*X, \end{array}$$

where *Y* = outcome and *X* = pain catastrophizing.

Intercept, slope, and explained variance for each equation are summarized in Table 2. The relationship between pain catastrophizing and pain interference and physical health decreased as pain intensity increased. For example, for mild pain levels, every 1-point increase in pain catastrophizing, which has a 0–36 range, was associated with a 0.23 point increase in pain interference, which has a 0–10 point range. In contrast, for moderate and severe pain, the effect of 1-point increase in pain catastrophizing was reduced to 0.16 and 0.07 points, respectively. A similar pattern emerged for Physical Functioning, Role Physical, and General Health. The relationship between 1-point increase in pain catastrophizing and 1-point decrease in the Mental Composite Score was comparable across the three pain groups. This is consistent with the moderation analyses in Table 1.

Table 2 also revealed that the variance of pain interference and physical health outcomes explained by pain catastrophizing differed across pain groups. As pain increased, especially for severe pain levels, there was a decrease in the variance explained by pain catastrophizing. In contrast, when mental health was the outcome variable, pain catastrophizing explained a comparable percentage of variance irrespective of pain levels.

## 4. Discussion

The present study aimed to explore whether the relationship between pain catastrophizing and important health outcomes was contextually determined by pain intensity. Previous studies had shown that pain catastrophizing is associated with pain interference and physical and mental health status across several pain populations above and beyond the contribution of pain intensity [7]. However, research had also revealed that features (i.e., areas activated in the brain) of pain catastrophizing might depend on pain intensity ratings [13], suggesting that the relationship between pain catastrophizing and pain-related outcomes may be somehow influenced by pain characteristics. Results in the present investigation provide partial support for the latter. Specifically, while pain catastrophizing contributed to all study outcomes after controlling for pain intensity, the strength of the relationship between pain catastrophizing and certain outcomes, namely, pain interference and physical health status, varied as a function of pain intensity levels. Support for the idea that pain intensity moderates the relationship between pain catastrophizing and health outcomes was only partial because moderation did not occur in the relationship between pain catastrophizing and mental health.

Results in the present study may have important theoretical and clinical implications for treatments of chronic pain, first, because the idea that pain intensity may moderate the relationship between pain catastrophizing and pain outcomes is new to existing psychological models of pain. For example, the fear-avoidance model of pain, one of the best-established psychological models of health in pain settings, argues that disability and distress occur as a result of an interpretation of pain as a catastrophe, which leads to fear and avoidance of activity [27, 28]. However, a review of research into fear-avoidance argued that the model should reconsider pain intensity as a primary factor for Physical Functioning because “high pain intensity is in itself a threatening experience that drives escape and avoidance” [12]. Consistent with this idea, our results suggest that the direct association between pain catastrophizing and Physical Functioning, as described in the fear-avoidance model, might in fact be moderated by pain intensity levels.

Results in the current investigation may also provide new insight into psychological interventions in chronic pain. Past research has shown that pain catastrophizing can be reduced [29] and that such changes lead to improved physical and mental health status of pain patients [30]. However, consistent with our findings, there is also evidence to suggest that the effectiveness of psychological interventions in pain settings might be contextually determined. For example, while changes in pain catastrophizing have been associated with improved physical and mental health status, the strength of this relationship is strongest for mental well-being [31]. In light of our findings, pain intensity might be the contextual factor explaining why a reduction in pain catastrophizing is mostly associated with improved mental health status. This finding is important because existent psychological treatments tend to ignore patients' pain intensity as a variable influencing treatment effectiveness [6, 32, 33]. Our results suggest that this practice might be adequate when attempting to improve mental well-being. However, pain intensity levels should not be overlooked when pain interference and physical disability are the outcomes. Specifically, it is possible that attempts to reduce pain interference and physical disability via pain catastrophizing will work best if pain is reduced first, especially when patients experience severe pain intensity.

An important finding in the present study was that pain intensity did not moderate the relationship between pain catastrophizing and mental health. Research has already evidenced that pain catastrophizing is more strongly associated with mental components of health when compared to physical outcomes [11]. Our results are consistent with this idea. However, the present study extends past research by showing that the contribution of pain catastrophizing on mental health status may be comparable across different pain levels. Studies have already demonstrated that pain intensity is more related to physical disability than to mental well-being [12]. Therefore, one possible interpretation of our results is that the disability levels of patients with severe pain are such that there is little room for psychological factors like pain catastrophizing to influence Physical Functioning. In contrast, because pain intensity has a weaker effect on mental health, the contribution of pain catastrophizing may remain unaltered irrespective of pain levels. Therefore, our results suggest that a reduction of pain intensity prior to psychological treatment might not be needed when mental well-being is the intervention outcome.

The present study is not without limitations. For example, the cross-sectional nature of our data prevents us from drawing causal inferences. The assessment of patients from two different recruitment sites may also be problematic. However, we addressed this by including the assessment site in the regression analyses. It is important to note that moderation occurred even after controlling for assessment site and other covariates of functioning, with the only exception of the Physical Functioning Scale. Finally, psychological factors other than pain catastrophizing were not explored in the present investigation, so our results cannot be generalizable to other psychological variables. Further research should explore whether our findings are replicated using important psychological factors in pain research, such as pain acceptance, fear of pain, pain self-efficacy, and pain vigilance.

In conclusion, while research has shown that pain catastrophizing affects outcomes [11], the present investigation shows that the strength of the relationship between pain catastrophizing and predominantly physical health outcomes may vary as a function of pain intensity levels. In contrast, correlations between pain catastrophizing and mental well-being appear to be comparable irrespective of pain levels. These results suggest that, while the relationship between pain catastrophizing and mental well-being might be well represented in existent models of pain, the role of pain intensity should be reconsidered in relation to pain interference and physical health outcomes. With regard to existing psychological interventions in pain settings, researchers are encouraged to test whether, for patients with severe pain levels, a reduction of pain intensity before changing pain catastrophizing is a recommendable practice when pain interference and Physical Functioning are the treatment outcomes. It is possible that such a personalized treatment helps maximize the beneficial effect of reducing pain catastrophizing. In light of our results, this practice might not be necessary when attempting to improve mental well-being.

## Supplementary Material

Table S1 shows a comparison of continuous variables of interest (i.e., age, pain characteristics, health status, and pain catastrophizing) across the two hospitals. Categorial variables (i.e., demographic characteristics and diagnosis of depression or anxiety) are compared in Table S2.

## Figures and Tables

**Table 1:** Hierarchical regression analysis for the prediction of pain interference, physical components of health, and the mental health composite.

	Pain interference		Physical Functioning		Role Physical		General Health		Mental Composite	
	*β*	Δ*R*^2^	*β*	Δ*R*^2^	*β*	Δ*R*^2^	*β*	Δ*R*^2^	*β*	Δ*R*^2^
Block 1		.465^c^		.226^c^		.261^c^		.217^c^		.116^c^
Pain intensity	.68^c^		−.48^c^		−.51^c^		−.47^c^		−.35^c^	
Block 2		.123^c^		.097^c^		.100^c^		.204^c^		.240^c^
Pain intensity	.46^c^		−.28^c^		−.31^c^		−.19^b^		−.04	
Pain catastrophizing	.42^c^		−.37^c^		−.38^c^		−.54^c^		−.58^c^	
Block 3		.021^c^		.014^a^		.019^b^		.022^b^		<.001
Pain intensity	.45^c^		−.27^c^		−.30^c^		−.17^b^		−.04	
Pain catastrophizing	.44^c^		−.40^c^		−.41^c^		−.56^c^		−.58^c^	
Pain intensity × Pain catastrophizing	−.15^c^		.13^a^		.15^b^		.16^b^		−.01	
Block 4		.011		.094^c^		.038^b^		.007		.079^c^
Pain intensity	.43^c^		−.17^b^		−.24^c^		−.14^a^		−.05	
Pain catastrophizing	.40^c^		−.31^c^		−.35^c^		−.51^c^		−.57^c^	
Pain intensity × pain catastrophizing	−.16^c^		.06		.11^a^		.15^b^		.03	
Recruitment site	−.05		.21^c^		.10		.08		−.09	
Age	−.12^a^		−.19^c^		.04		.08		.16^b^	
Sex	−.04		−.06		<.01		<.01		−.05	
Job status	−.06		.12^a^		.22^c^		<.01		−.07	
Marital status	−.02		.04		−.06		−.03		.10	
Educational level	−.05		.01		−.07		.04		.02	
Psychopathology	.07		<.01		<.01		−.11		−.23^c^	
Pain duration (years)	<.01		.06		.05		−.03		−.04	
Total *R*^2^		.620		.431		.418		.450		.435

*Note*. Beta values are standardized. Reported *ΔR*^2^ is adjusted and represents the change in *R*^2^ for each block. Pain intensity and pain catastrophizing were centered. The covariates were entered in the last block to explore whether the interaction term in block 3 remained significant when controlling for important covariates of health status. Categorical variables in block 4 were coded as follows: recruitment site (0 = tertiary pain clinic; 1 = primary care), sex (0 = men; 1 = women), job status (0 = not working; 1 = working), marital status (0 = not married; 1 = married), educational level (0 = less than 12 years of education; 1 = more than 12 years of education), and psychopathology (0 = no diagnosis of depression or anxiety; 1 = diagnosis of depression or anxiety).

^a^ *p* < .05.

^b^ *p* < .01.

^c^ *p* < .001.

**Table 2:** Slopes, intercepts, explained variance, and bivariate associations between pain catastrophizing and each outcome across pain categories.

	Mild pain (*n* = 80)	Moderate pain (*n* = 103)	Severe pain (*n* = 71)
*Pain interference*			
Intercept	0.90	3.30	5.95
Slope	0.23	0.16	0.07
*R* ^2^	.49	.28	.07
*r*	.70^c^	.53^c^	.29^a^
*Physical functioning*			
Intercept	85.01	71.99	40.33
Slope	−2.18	−1.39	0.35
*R* ^2^	.28	.15	.01
*r*	−.53^c^	−.39^c^	−.18
*Role Physical*			
Intercept	81.42	58.16	43.97
Slope	2.47	1.03	−0.91
*R* ^2^	.40	.09	.09
*r*	−.63^c^	−.29^b^	−.30^a^
*General Health*			
Intercept	75.25	69.05	41.96
Slope	−2.05	−1.73	−0.57
*R* ^2^	.45	.35	.05
*r*	−.67^c^	−.59^c^	−.23
*Mental Composite Score*			
Intercept	53.02	54.19	51.16
Slope	−0.80	−0.92	−0.80
*R* ^2^	.26	.31	.24
*r*	−.51^c^	−.55^c^	−.49^c^

Intercepts, slopes, and explained variances refer to the regression lines. Bivariate associations were calculated using Pearson correlations. Bivariate associations and slopes differ because study variables were not standardized.

^a^ *p* < .05.

^b^ *p* < .01.

^c^ *p* < .001.
