# Supplementary material for: Pain Catastrophizing and Its Relationship with Health Outcomes: Does Pain Intensity Matter?
Source: Pain Res Manag. 2017 Feb 28;2017:9762864. doi: 10.1155/2017/9762864 (PMC5350380; doi:10.1155/2017/9762864)
Supplement: Supplementary file 1 — Table S1 shows a comparison of continuous variables of interest (i.e., age, pain characteristics, health status, and pain catastrophizing) across the two hospitals. Categorial variables (i.e., demographic characteristics and diagnosis of depression or anxiety) are compared in Table S2. [file 9762864.f1.docx]

Table S1. Comparison of continuous variables of interest (i.e., age, pain characteristics, health status, and pain catastrophizing) in two hospitals.

|  | Pain Unit  (n = 164) | Primary Care  (n = 90) | *t* |
| --- | --- | --- | --- |
| Age | 54.34 (12.67) | 49.12 (12.65) | 3.14^b^ |
| Average pain intensity | 5.96 (1.70) | 4.18 (1.81) | 7.79^c^ |
| Pain interference | 5.97 (2.30) | 3.63 (2.55) | 7.44^c^ |
| Pain duration (years) | 9.65 (8.91) | 8.57 (9.21) | 0.89 |
| Physical Functioning | 42.02 (23.04) | 70.22 (24.64) | -9.07^c^ |
| Role Physical | 35.94 (24.60) | 59.38 (24.11) | -7.31^c^ |
| General Health | 39.51 (19.82) | 56.80 (20.77) | 6.51^c^ |
| Mental Composite Score | 40.39 (11.76) | 44.05 (10.83) | -2.42^a^ |
| Pain catastrophizing | 15.55 (7.43) | 9.29 (6.78) | 6.61^c^ |

^a^*p* < .05

^b^*p* < .01

^c^*p* < .001

Table S2. Comparison of categorical variables of interest (i.e., demographic characteristics and diagnosis of depression or anxiety) in two hospitals.

|  | Pain Unit  (n = 164) | Primary Care  (n = 90) | *χ^2^* |
| --- | --- | --- | --- |
| Men (%) | 33.1 | 46.7 | 4.51^a^ |
| Married (%) | 53.7 | 42.2 | 3.04 |
| >12 years of education (%) | 67.1 | 85.6 | 10.22^b^ |
| Working (%) | 25.6 | 63.3 | 34.77^c^ |
| Psychopathology (%) | 32.7 | 12.4 | 12.53^c^ |

^a^*p* < .05

^b^*p* < .01

^c^*p* < .001
